# Research Progress on Nicotine Control Methods in Tobacco Products

**DOI:** 10.3390/molecules31142465

**Published:** 2026-07-15

**Authors:** Xu Xu, Haoxuan Song, Shuaiyu Chen, Yu Wang, Hong Ye

**Affiliations:** 1 Research Center for Innovative Applications in Health Security, Beijing Technology and Business University, Beijing 100048, China; xux@th.btbu.edu.cn (X.X.); sychen200301@163.com (S.C.); 2 Key Laboratory of Geriatric Nutrition and Health (Beijing Technology and Business University), Ministry of Education, Beijing 100048, China; s717501@163.com; 3 School of Advanced Materials and Future Technologies, Beijing Technology and Business University, Beijing 100048, China; 13856129491@163.com

**Keywords:** nicotine, tobacco products, controlled release

## Abstract

This paper systematically reviews recent research progress on nicotine control methods in tobacco products, with a focus on various strategies including the regulation of internal tobacco components (such as organic acids, pH, polyols, and moisture content) and the application of external functional materials (e.g., bacterial cellulose-based sustained-release systems and MOF composites). The mechanisms and control efficacy of these approaches are elaborated, along with a comparative analysis of their applicable conditions and limitations. Furthermore, emerging research directions in the precise control of nicotine release are discussed, including multi-factor coupling regulation, material structure design, and release kinetic modeling. By systematically examining the strengths and weaknesses of existing technologies, product-specific and condition-dependent internal control strategies are summarized for different tobacco products based on the available evidence. Finally, future prospects are outlined, including the development of intelligent sustained-release materials, the construction of multi-path synergistic regulation systems, and the integration of toxicological assessments to achieve comprehensive optimization of product quality and health risks. This review aims to provide a mechanistic basis for the rational design of nicotine release control strategies in tobacco products.

## 1. Introduction

Nicotine release during puffing is a key determinant of nicotine delivery, sensory impact, and product acceptability in tobacco products [1,2,3,4,5,6]. Nicotine (C_10_H_14_N_2_, chemically 3-(1-methylpyrrolidin-2-yl)pyridine) is one of the major alkaloids in tobacco and an important constituent of tobacco leaves; its content significantly influences cigarette quality. As a key component of mainstream smoke, nicotine is also closely associated with smokers’ sensory experience. Excessively high or rapid nicotine release can cause strong impact and irritation, leading to oral discomfort and a short-lived sensation that does not meet sustained physiological needs. Conversely, insufficient or delayed nicotine release may weaken sensory intensity and reduce perceived product quality. Notably, several studies [7,8,9,10] have shown that puff-by-puff nicotine release first increases and then decreases over the puff sequence, suggesting that stable puff-by-puff nicotine delivery remains difficult to maintain. Because puff-by-puff nicotine release behavior is a critical factor affecting the sensory quality of tobacco products, the effective regulation of nicotine release has become an important research focus in the tobacco industry.

In tobacco products, nicotine primarily exists in three forms: free-base, monoprotonated, and diprotonated nicotine. These forms differ in their bioavailability and sensory effects. Free-base nicotine is mainly present in the gas phase. It can be absorbed through the oral mucosa and act on the central nervous system, producing a stronger smoke impact [11,12,13,14,15,16,17]. In contrast, protonated nicotine is not readily absorbed orally [18], has low bioavailability, and serves as the main storage form of nicotine. Thus, a higher free-base nicotine content in cigarettes generally leads to greater free-base nicotine release in smoke [19,20,21]. Notably, during the puffing of heated cigarettes, protonated nicotine can be thermally converted into the free-base form [22,23], further influencing nicotine release behavior and bioavailability. Moreover, nicotine content in tobacco leaves is significantly and positively correlated with smoke tar yield [24,25,26,27,28,29,30]; therefore, precise regulation of nicotine release may indirectly contribute to the synergistic control of tar and other harmful constituents.

Extensive research has been devoted to regulating nicotine release, leading to a variety of technical pathways, including compositional modification and material-based intervention. However, reviews that synthesize these studies from the perspective of nicotine release control remain scarce. The few related reviews that are available mainly focus on nicotine forms, detection methods, or biological effects. Han et al. [31] reviewed the differences in release behavior and physiological effects among different nicotine forms, including sensory characteristics, absorption, toxicity, and dependence potential. For instance, Lu et al. [32] summarized nicotine content and speciation in e-liquid and aerosol, but their review focused on analytical techniques rather than controlled release. Tao et al. [33] reviewed pretreatment and analytical methods for nicotine detection in diverse and complex matrices but did not address controlled release. Yang et al. [34] reviewed the association between genetic polymorphisms in the dopaminergic system and nicotine dependence, but controlled release methods were not discussed. Consequently, an integrated synthesis of the mechanisms and methods for nicotine release regulation is still lacking. This gap limits the theoretical and technical support available for developing and upgrading nicotine controlled-release technologies in tobacco products. To address this gap, this review provides an overview of recent research on nicotine release regulation in tobacco products. Where available, reported quantitative outcomes are summarized, and the mechanisms, applicable conditions, and limitations of different nicotine release control strategies are compared. It also analyzes the strengths and limitations of existing technologies and discusses current challenges and future directions. The overall aim is to provide a theoretical basis for constructing precise nicotine controlled-release systems. This review further proposes a synergistic controlled-release framework of “fundamental regulation–precise intervention” based on internal control strategies and external functional materials. It aims to contribute to the limited integrated literature in this field. Literature was searched using Web of Science and CNKI with topic-based searches.

## 2. Core Principles Governing Nicotine Speciation and Release Behavior

**Figure 1:**
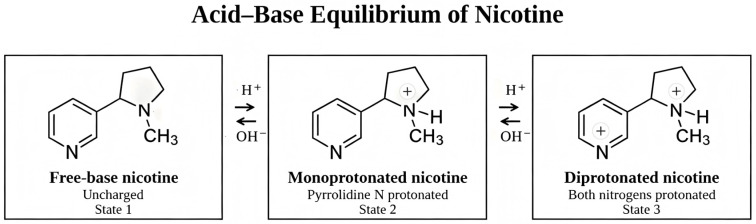
Three species of nicotine.

Nicotine contains two tertiary amine groups in its molecular structure and exhibits weak basicity, with pKa1 = 3.12 for the pyridine ring and pKa2 = 8.02 for the pyrrolidine ring [35]. Within the pH range of tobacco systems, nicotine is readily protonated to form monoprotonated or diprotonated nicotine salts, and its speciation is mainly determined by the system pH. Accordingly, nicotine exists in three chemical forms [36,37,38], and the chemical structures of these nicotine species are shown in Figure 1.

Existing studies have shown that the distribution of nicotine species in different tobacco products is largely regulated by system pH [39]. In combustible cigarettes, the free-base form of nicotine becomes detectable when the pH exceeds 6.0, with the free-base nicotine fraction (αfb) being approximately 1.5% [12]. When the pH exceeds 7.4, αfb increases to approximately 30%; when the pH exceeds 7.8, αfb reaches approximately 50% [19]. Several studies have also measured the free-base nicotine fraction in commercially available cigarettes. Duan et al. [40] tested two commercial cigarette brands and reported αfb values of 14.06% and 11.28%, respectively. Wu et al. [41] tested 38 commercial cigarette brands and reported αfb values ranging from 29% to 88%. Xu et al. [42] tested 17 commercial cigarette brands and found that αfb ranged from 12.9% to 38.9%.

For heated cigarettes, tests of 14 commercially available heated tobacco products showed that αfb varied across different puffing regimes. Under the ISO regime, αfb ranged from 25.42% to 37.62%, whereas under the HCI regime, it ranged from 11.06% to 23.38% [43].

In e-liquids, nicotine speciation is also closely related to pH. El-Hellani et al. [44] found that αfb was 2.5% at an e-liquid pH of 5.16, whereas it increased to 97.7% when the pH reached 9.66.

Overall, the free-base nicotine fraction is approximately 10–88% in combustible cigarettes and approximately 11–38% in heated cigarettes under different puffing regimes. In e-cigarettes, the free-base nicotine fraction can vary from approximately 2% to 98% through regulation of e-liquid pH or addition of organic acids, while the remaining nicotine mainly exists in the monoprotonated form. It should be noted that only a limited number of studies have theoretically calculated or indirectly estimated the distribution of all three nicotine species. Most published studies report only free-base nicotine and its proportion in total nicotine. Therefore, the quantitative distribution of the three nicotine species requires further investigation.

In addition, after cigarette combustion, nicotine is mainly present in the particle phase of mainstream smoke, predominantly in the monoprotonated form, while a certain proportion exists as free-base nicotine. The diprotonated form usually accounts for a relatively small proportion because of the pH conditions of cigarette smoke.

In combustible cigarettes and non-combustible tobacco products, including heated cigarettes, e-cigarettes, and oral tobacco products, nicotine release behavior is a complex process governed by multiple coupled factors. First, nicotine is weakly basic and can be neutralized by organic or inorganic acids to form nicotine salts; its release is therefore closely associated with the surrounding acid–base conditions and, consequently, with the compositional makeup of tobacco leaves and e-liquids. Second, under the non-steady-state thermal environment generated during combustion or heating, different nicotine species can interconvert among the chemical forms shown in Figure 1, resulting in dynamic shifts in species distribution. Finally, nicotine species continuously undergo phase partitioning between the solid/gas or liquid/gas phases.

Based on the nicotine release characteristics described above, nicotine release can be regulated through the following mechanistic pathways:

- Chemical speciation equilibrium: By modulating system pH and the type and content of organic acids, the equilibrium between free-base and protonated nicotine species can be shifted. This shift directly affects nicotine bioavailability and sensory irritation and therefore serves as the core chemical basis for nicotine controlled release;
- Pyrolysis/atomization behavior: Factors such as moisture content and polyol content in the tobacco system directly influence combustion/heating temperature and tobacco pyrolysis kinetics, thereby altering both the total amount and rate of nicotine release;
- Gas–liquid phase partitioning: The aerosol matrix, such as glycerol and propylene glycol, influences the gas–liquid partition coefficient of nicotine, thereby altering its transfer efficiency into smoke and release stability;
- Diffusional mass transfer process: The porous structure and adsorption sites of external functional materials can retard nicotine release through diffusion retardation and physical adsorption, thereby improving puff-by-puff release stability;

Based on the core control principles outlined above, existing nicotine controlled-release strategies can be categorized into two groups: internal control strategies, which modulate nicotine speciation and release behavior in situ by altering tobacco constituents and processing parameters; and external controlled release via functional materials, which intervenes in nicotine release and mass transfer through mechanisms such as physical or chemical adsorption. Precise nicotine controlled release relies on the synergistic interplay between internal chemical regulation and external mass-transfer intervention; a single approach alone is insufficient to meet the practical demand for stable release.

## 3. Internal Control Mechanisms for Nicotine Release Based on Tobacco Constituents and Formulation Parameters

Tobacco constituents and formulation parameters are fundamental to the internal control of nicotine release. The core mechanism involves modulating nicotine speciation, pyrolysis efficiency, and transfer processes in situ by altering the chemical environment and thermal behavior of the tobacco system. These approaches offer operational simplicity, low cost, and ease of industrialization, and therefore represent the mainstream control strategies currently applied in tobacco production [32,33,34,35,45].

### 3.1. Organic Acid- and pH-Mediated Regulation of Nicotine Speciation

Organic acids are important chemical constituents of tobacco [45]. Together with system pH, they are key factors governing nicotine speciation and represent one of the most mechanistically well-characterized and widely applied pathways for nicotine controlled release [14,46]. Because nicotine is weakly basic, it can be neutralized by organic or inorganic acids to form nicotine salts. This process drives the conversion of free-base nicotine to protonated species, thereby reducing the proportion of free-base nicotine and diminishing smoke irritation. Conversely, increasing the system pH promotes the conversion of protonated nicotine to the free-base form, thereby enhancing smoke impact [40]. Based on this chemical equilibrium principle, the type and amount of organic acids and the regulation of system pH have become two direct technical approaches for nicotine controlled release.

#### 3.1.1. Differential Regulation of Nicotine Release by Organic Acids

Organic acids are important internal constituents in tobacco and can be divided into three groups based on volatility: volatile (e.g., acetic and propionic acid), semi-volatile (e.g., lactic acid), and non-volatile (e.g., citric, tartaric, and malic acid). These three classes differ substantially in their content in tobacco, thermal migration behavior, and control effects on nicotine release [47]. Non-volatile organic acids account for approximately 10% of the total leaf mass, modulating tobacco acid–base balance and enhancing smoke body. Volatile organic acids constitute only 0.1–0.2% of the leaf mass, yet they can directly enter the smoke aerosol during tobacco combustion or heating and react in situ with free-base nicotine [48,49]. Notably, the control effects of both non-volatile and volatile organic acids on nicotine release are strongly influenced by the mode of addition, site of action, and product type.

Regarding the mode of addition, Wang et al. [50] prepared three types of filter rods for conventional combustible cigarettes using different non-volatile organic acid addition methods. In the first method, filter rods were dip-coated in a citric acid solution, followed by freeze-drying to precipitate citric acid onto the cellulose acetate fibers. The second method involved injecting citric acid solution into the filter rods and then drying. The third method incorporated citric acid granules directly into the filter rods. The results showed that the first method was more effective in nicotine control than the other two: without altering the physical properties of the cigarette, freeze-drying with 5 mg citric acid per cigarette reduced free-base nicotine by 36.8%, whereas the other two methods exerted minimal effects. This superior performance is attributed to the uniform distribution of citric acid achieved through freeze-drying, which effectively increases its specific surface area and enhances its capacity to adsorb nicotine.

In heated tobacco products, semi-volatile lactic acid has also been shown to provide effective nicotine control. He et al. [10] prepared a tobacco core material from blended tobacco powders and loaded it with three acid flavorants: acetic acid, propionic acid, and lactic acid. They observed that, as the addition level of each flavorant increased, the release of both free-base nicotine and total nicotine in the aerosol showed a clear decreasing trend, with lactic acid producing the greatest reduction. At a lactic acid loading of 3.0%, free-base nicotine and total nicotine release decreased by 75% and 20%, respectively.

However, some studies have found that the addition of non-volatile acids does not produce a meaningful nicotine-controlling effect. For instance, in a study of heated tobacco products, Qin et al. [51] observed that adding three non-volatile organic acids—citric acid, tartaric acid, and malic acid—at levels of 0.5–2% produced no appreciable change in the proportion of free-base nicotine in smoke. In contrast, adding volatile organic acids, such as levulinic acid, propionic acid, and acetic acid, led to a clear decrease in the proportion of free-base nicotine. Among these acids, acetic acid and levulinic acid showed better performance; in particular, adding 2% levulinic acid reduced the proportion of free-base nicotine in smoke from 5.62% to 1.76%. The authors attributed this effect to the ability of volatile organic acids to transfer directly into the smoke aerosol during tobacco combustion and react with free-base nicotine to form nicotine salts.

Although the findings of these studies may seem inconsistent, they indicate that the ability of an organic acid to control nicotine release depends on a combination of factors. For instance, because of its relatively high boiling point, citric acid exerts a noticeable nicotine-controlling effect mainly in combustible cigarettes, where the high combustion temperature enables it to react directly with free-base nicotine in smoke. Under non-combustible heating conditions, typically around 350 °C, the volatility of citric acid is limited, making it much less effective. Lactic acid, as a semi-volatile organic acid, is more volatile than citric acid and can still interact with free-base nicotine under non-combustible heating conditions. Furthermore, lactic acid contains both a carboxyl group (–COOH) and a hydroxyl group (–OH), whereas acetic acid and propionic acid contain only a single carboxyl group. This bifunctional structure may provide additional control advantages compared with compounds containing only a single carboxyl group, thereby improving nicotine control. Volatile organic acids, such as acetic acid, can interact more readily with free-base nicotine in heated cigarettes, but their high volatility may also lead to partial loss. Overall, lactic acid is one of the few organic acids compatible with both conventional combustible cigarettes and heated products, making it a favorable option for nicotine control.

The phenomena observed in the above studies can be further explained from a theoretical perspective. Duell et al. [52] conducted theoretical derivation and experimental validation of nicotine speciation and its control principles in tobacco products. By analyzing various bottled e-liquids, they examined the proportion of free-base nicotine and its relationship with organic acid content and nicotine release behavior. The results demonstrated that the free-base nicotine fraction is jointly governed by three factors: the molar ratio of organic acid to nicotine, the dissociation capacity of the organic acid, and the solvent environment of the reaction system. The study traced the evolution of product design in conventional cigarettes over nearly 400 years and in e-cigarettes over the past two decades, revealing that both product categories have followed a similar technological trajectory. Specifically, conventional cigarettes evolved from strongly irritating air-cured tobacco with initially high free-base nicotine (≥0.5) to products with a free-base nicotine fraction stabilized at approximately 0.1 through process modifications. Similarly, e-cigarettes transitioned from early-generation pure free-base nicotine systems (≈1) to nicotine salt formulations through organic acid addition, ultimately reducing the free-base nicotine fraction to approximately 0.1. This cross-category pattern suggests that organic acid-mediated nicotine speciation regulation is an effective nicotine controlled-release strategy that has been supported by long-term market experience in the tobacco industry. The study further quantified the effect of the organic acid-to-nicotine molar ratio on the free-base nicotine fraction. When the molar ratio of benzoic acid to nicotine approached 1:1, the free-base nicotine fraction stabilized between 0.11 and 0.13. When the acid-to-nicotine ratio substantially exceeded 1, the free-base nicotine fraction could approach zero; in the absence of added organic acid, the free-base nicotine fraction remained close to 1. Based on the above studies, organic acids with relatively low boiling points, such as lactic acid and benzoic acid, can achieve favorable nicotine control under both combustion and heated conditions, making them preferred candidates for nicotine regulation among organic acids.

Although the studies described above demonstrate that nicotine release is influenced by organic acids, in-depth mechanistic analyses remain limited. A thorough understanding of the mechanisms by which organic acids affect nicotine release requires a comprehensive evaluation of multiple factors, including the pKa values, molecular structures, and interaction types of the organic acids, as well as environmental conditions and changes in the free-base nicotine fraction at different acid addition levels.

Beyond studies on specific organic acid addition, some investigations have shifted the focus from individual acid species to changes in pH, an overall indicator of acidity, to examine nicotine release behavior.

#### 3.1.2. Control of Nicotine Release by pH

System pH is a key indicator governing nicotine speciation. The fraction of free-base nicotine in total nicotine is significantly and positively correlated with the pH of the smoke system, making pH a direct regulatory target for nicotine controlled release [14,46]. Chen et al. [14] measured smoke particulate matter pH, total nicotine, and free-base nicotine content in nine Chinese cigarette brands and found that the free-base nicotine fraction was correlated with smoke particulate matter pH: the lower the pH, the lower the proportion of free-base nicotine in total nicotine. Other studies [46] have reported a similar pattern, showing that the free-base nicotine fraction is closely linked to cigarette pH.

Based on these findings, researchers began to use acid or base addition to adjust pH for nicotine control, thereby confirming the directional effect of pH variation on the free-base nicotine fraction. Duan et al. [40] treated cigarette cut tobacco with different amounts of NaOH solution (0.5 mol/L) and hydrochloric acid (0.2 mol/L). The results showed that the free-base nicotine fraction decreased as hydrochloric acid addition increased. After 160 mL of hydrochloric acid was added, the pH of the cut tobacco decreased from 5.19 in the untreated sample to 4.23, and the free-base nicotine fraction decreased from 14.06% to 6.96%. Conversely, the free-base nicotine fraction increased with NaOH addition. When 267 mL of NaOH solution was added, the pH of the cut tobacco increased from 5.28 to 6.71, and the free-base nicotine fraction increased from 11.28% to 17.40%. These results demonstrate that both organic and inorganic acids can be used to control nicotine release and that acids and bases can be combined to regulate nicotine release.

Subsequent studies expanded the sample size to verify whether this pattern was generally applicable to commercially available combustible cigarette products. Wu et al. [41] measured total nicotine, free-base nicotine, bound nicotine content, and smoke particulate matter pH in 38 cigarette brands and analyzed the relationship between smoke particulate matter pH and nicotine speciation. The correlation coefficient between free-base nicotine content and smoke particulate matter pH was 0.846, indicating a highly significant positive correlation. Xu et al. [42] further extended the scope of smoke pH measurement beyond previous studies that focused only on smoke particulate matter pH. For conventional combustible cigarettes, they simultaneously measured the pH of the gas phase, whole smoke, and particulate matter of mainstream smoke. Their results confirmed that the free-base nicotine fraction is correlated not only with smoke particulate matter pH but also with whole-smoke pH, thereby providing a more complete mechanistic picture of how the acid–base environment of the entire smoke system influences nicotine speciation.

The puffing regime also affects free-base nicotine release. Liu et al. [43] tested 14 cigarette samples under the International Organization for Standardization (ISO) regime and the Health Canada Intense (HCI) puffing regime. Under the ISO regime, nicotine content in smoke particulate matter ranged from 0.41 to 0.59 mg/cig, free-base nicotine content ranged from 0.11 to 0.22 mg/cig, and the free-base nicotine fraction ranged from 25.42% to 37.62%. Under the HCI regime, the corresponding values were 1.17–1.69 mg/cig for nicotine, 0.15–0.39 mg/cig for free-base nicotine, and 11.06–23.38% for the free-base nicotine fraction. Under both regimes, the free-base nicotine fraction was significantly and positively correlated with smoke particulate matter pH.

The aforementioned studies were conducted in conventional combustible cigarette systems and collectively validate the full-chain principle of pH-mediated nicotine release control. Research on different categories of tobacco products has further shown that the application of this core principle varies according to the nicotine absorption pathway. Unlike combustible cigarettes and heated cigarettes, in which nicotine is inhaled through the respiratory tract, oral tobacco products do not involve combustion or atomization; instead, nicotine is absorbed entirely through the oral mucosa. Because only free-base nicotine can penetrate oral mucosal epithelial cells, the regulatory logic of pH for oral tobacco products differs fundamentally from that for cigarettes. Seidenberg et al. [53] compared the product characteristics of American snus and Swedish snus marketed in the United States and elucidated the critical influence of pH on nicotine release and absorption potential. Based on 2014 industry report data from the Massachusetts Department of Public Health, the study analyzed 14 American-style and 10 Swedish-style products. The results showed that the pH of Swedish snus (median, 8.68) was significantly higher than that of American-style products (median, 6.54). Correspondingly, the free-base (unionized) nicotine concentration (6.52 mg/g vs. 0.52 mg/g) and the free-base nicotine fraction of total nicotine (81.8% vs. 3.17%) were markedly higher in Swedish snus. This study confirmed that pH is a key factor governing nicotine absorption efficiency in oral tobacco products.

Together, these studies provide clear directions for precise pH control in different tobacco product types. For combustible cigarettes and heated cigarettes, lowering the system pH can reduce smoke irritation, whereas for oral tobacco products, raising the pH can enhance oral nicotine absorption efficiency. pH-mediated regulation of nicotine speciation by organic acids constitutes the core chemical basis of internal controlled release.

### 3.2. Regulation of Nicotine Release by Polyols

Glycerol and propylene glycol are low-boiling-point polyols that are generally added to tobacco products during manufacturing to maintain tobacco moisture, retard oxidation, and modulate flavor.

In conventional combustible cigarettes, polyol humectants are typically added at 0.5–2% of the cut tobacco mass. Their primary functions are to improve the moisture-retention properties of cut tobacco, regulate combustion behavior, and optimize sensory quality, while exerting only a minor and indirect influence on nicotine release. Under most combustion conditions, glycerol and propylene glycol undergo extensive thermal decomposition, oxidation, and combustion reactions, with only a small fraction entering mainstream smoke. Their influence on nicotine release is generally mediated indirectly through changes in moisture content, pore structure, peak combustion temperature, and smoke particulate matter formation, and this topic has received limited research attention. Liu et al. [54] added various humectants, including propylene glycol, glycerol, humectant No. 27664, xylitol, and sorbitol, to cigarette cut tobacco at mass fractions of 1% and 2%. The samples were equilibrated for 48 h and then used to prepare cigarettes for evaluating the effects of different humectants on the chemical composition of cigarette smoke. The study found that most humectants increased the free-base nicotine content in smoke, whereas only 1% glycerol reduced free-base nicotine content; however, the original report did not specify the magnitude of this reduction.

In heated cigarettes, glycerol and propylene glycol no longer serve merely as auxiliary humectants; instead, they function as the core atomization matrix in the formulation, with typical addition levels ranging from approximately 10% to 30% of the core material or e-liquid mass. During heating and atomization, they generate a large quantity of stable aerosol. The underlying principle can be understood as follows: polyols can form strong hydrogen bonds with nicotine molecules and function as carriers that efficiently transport nicotine into smoke during atomization, thereby enhancing the transfer efficiency of nicotine from the tobacco matrix to the smoke phase. Because glycerol and propylene glycol contain different numbers of hydroxyl groups, their binding capacities with nicotine also differ. Consequently, varying the content of glycerol or propylene glycol can modulate the amount of nicotine released, and their combined formulation can regulate both the rate and total amount of nicotine release over a considerable range.

Tang et al. [55] sprayed aqueous glycerol solutions onto 50 g tobacco samples at 5%, 10%, and 15% of the cut tobacco mass to investigate the effect of glycerol on smoke release under heated conditions. They found that, as the heating temperature increased from 200 °C to 400 °C, nicotine release increased by 61–115%. As the glycerol addition level was gradually raised to 10%, nicotine release increased by 10–46%, but no appreciable change was observed when glycerol addition exceeded 10%. Hu et al. [56] reported similar findings when studying the effect of glycerol on smoke release. They also added propylene glycol to tobacco samples at 5%, 10%, and 15% and found that propylene glycol had no appreciable effect on nicotine release, in clear contrast to glycerol. These results indicate that glycerol exerts a greater influence on nicotine release, which may be attributable to its larger number of hydroxyl groups and stronger interactions with nicotine. Zhu et al. [57] further examined the effect of glycerol on nicotine in heated cigarettes by preparing tobacco sheets with glycerol application ratios of 5–30%. The study found that, as the glycerol addition ratio increased, the proportion of free-base nicotine relative to total nicotine also increased appreciably, indicating that glycerol can effectively promote nicotine release and that the two exhibit favorable synergistic behavior.

Gupta et al. [58] compared the effects of different puffing regimes on the aerosol release characteristics of e-cigarettes, providing important methodological support for regulating nicotine release behavior using polyols such as propylene glycol and glycerol as the aerosol matrix. The results demonstrated that puffing waveform, puffing volume, puffing duration, and puffing frequency all have pronounced effects on nicotine release and release stability. Among these parameters, the square-wave puffing regime provides a stable airflow rate, ensures consistent operation of the atomization device, and yields markedly higher total nicotine release than bell-shaped and triangular wave regimes, with an increase of approximately 0.95 mg in cumulative nicotine release over 400 puffs. Further investigation revealed that, when the puffing volume was 55–70 mL and the puffing duration was 3–4 s, the relative composition of nicotine and polyols in the aerosol remained highly stable, with fluctuations below 10%. This finding indicates a pronounced quantitative coupling between polyol consumption as the aerosol matrix and the nicotine atomization process. This study clarifies the carrier role and control mechanism of polyols in the nicotine release process and provides an experimental basis for the optimized design of e-cigarette atomization systems.

Taken together, most studies indicate that glycerol promotes nicotine release, whereas research on the effects of other polyols on nicotine release remains limited and requires further investigation. Among the tobacco product types studied, heated cigarettes have received the most attention, and the release-promoting effect of glycerol is more pronounced in these studies; research on other tobacco product types remains relatively scarce. Thus, glycerol addition represents a typical strategy for promoting nicotine release in heated cigarettes. Through its carrier effect, glycerol enhances nicotine transfer efficiency and serves as a key approach for internal regulation in heated cigarettes.

### 3.3. Effect of Moisture Content on Nicotine Release

Moisture content in cut tobacco is a key parameter, typically ranging from 11.00% to 13.50% [59]. During processing, higher moisture content improves the shatter resistance of cut tobacco and reduces breakage, making it an important quality indicator for cigarettes. Moreover, the moisture content of cut tobacco also exerts a notable influence on nicotine release.

For conventional combustible cigarettes, moisture content shows a clear negative correlation with nicotine release: as moisture content increases, nicotine release decreases. This effect is attributed to water vaporization during combustion, which absorbs heat, lowers the peak combustion temperature, and suppresses the thermal evaporation and volatilization of nicotine.

In a study of conventional combustible cigarettes, Li et al. [60] equilibrated cigarette samples for 48 h under different relative humidity conditions to obtain samples with varying moisture contents and investigate the influence of cut tobacco moisture content on smoke components. The study found that nicotine release decreased as moisture content increased, whereas draw resistance, smoke moisture content, tar, and CO levels all increased. This phenomenon occurs because higher moisture content consumes more heat during water vaporization. As a result, the combustion temperature decreases, combustion becomes less complete, and the number of puffs increases. These changes ultimately lead to lower nicotine levels and higher yields of tar and carbon monoxide (CO). Using the same sample preparation method, Sun et al. [61] also confirmed that nicotine content decreased and CO levels increased markedly with increasing moisture content. Zhang et al. [62] extended the range of equilibration humidity used in previous studies and employed simultaneous thermal analysis to examine the combustion properties of cut tobacco equilibrated at different humidities. The results demonstrated that moisture content exerts a pronounced effect on the combustion and pyrolysis behavior of cut tobacco. Excessively high moisture content reduces the combustion rate, thereby affecting nicotine release in smoke, whereas excessively low moisture content causes overly rapid combustion and adversely affects the sensory quality of the cigarette.

Beyond combustible cigarettes, studies have also examined the influence of cut tobacco moisture content on nicotine release in heated cigarettes. Li et al. [63] equilibrated cigarette samples for 96 h at 22 ± 1 °C under 40%, 50%, and 60% relative humidity to prepare samples with different moisture levels for testing. The results showed that nicotine release in smoke from cigarettes equilibrated at 50% relative humidity was slightly higher than that from samples equilibrated at 40% and 60% relative humidity. The study did not identify a definitive reason for the existence of an optimal nicotine release range. However, based on the properties of nicotine and the principles of heat-induced release, the following explanation can be inferred: in a heating system without open-flame combustion, moisture primarily influences heat-transfer efficiency and the desorption and migration capacity of nicotine. When the moisture content is too low, heat transfer within the tobacco matrix is uneven, making it difficult for nicotine to be released from its bound state. When the moisture content is too high, water occupies the pores and impedes aerosol entrainment, thereby reducing the transfer efficiency of nicotine into the smoke phase.

In summary, cut tobacco moisture content exerts a clear influence on nicotine release. For most conventional combustible cigarettes, nicotine release decreases as moisture content increases. However, in practical production, the risks of incomplete combustion caused by elevated moisture content and the potential reduction in product quality stability caused by moisture evaporation must be considered.

### 3.4. Internal Control Strategies for Nicotine Release

Section 3.1, Section 3.2 and Section 3.3 have discussed in detail the control mechanisms of three internal factors: organic acids and pH, polyols, and moisture content. Building on these discussions, this section provides a comparative analysis to further clarify the functional characteristics and applicable boundaries of these three approaches, thereby offering a broader basis for subsequent strategy selection.

In terms of stage of action, the three approaches occupy distinct control positions within the nicotine release process. Organic acids and pH act directly on the chemical speciation of nicotine by altering the ratio of free-base to bound nicotine through the protonation–deprotonation equilibrium; they therefore represent front-end factors that influence the potential for nicotine release. Polyols primarily influence the transfer efficiency of nicotine from the matrix to the aerosol and therefore represent a transport-stage intervention in the release process. Moisture content indirectly affects the thermal release of nicotine by altering the temperature field and heat-transfer pathways of the system. Its influence spans the entire release process but lacks chemical selectivity. These three approaches therefore target different stages of the nicotine release process, forming a control chain that extends from front-end regulation to terminal transfer and from chemical to physical mechanisms.

In terms of product applicability, the three approaches differ notably in their scope. First, organic acid and pH regulation shows relatively broad cross-category applicability, with potential applications in combustible cigarettes, heated cigarettes, and e-cigarettes, although its effectiveness is strongly influenced by the volatility of the organic acids used. Volatile organic acids can act effectively across different product types, whereas low-volatility organic acids, such as citric acid, are more effective in high-temperature combustion systems. Lactic acid is a notable modulator because it is suitable for both combustible and non-combustible tobacco products. Second, the control effect of polyols is highly product-type dependent. In heated cigarettes and e-cigarettes, glycerol serves as a key carrier that promotes nicotine release. Finally, the effect of moisture content is not monotonic across product types. In conventional cigarettes, high moisture content reduces nicotine release, whereas in heated cigarettes, an optimal nicotine release range may exist. This indicates that product type should be a primary consideration when selecting internal control strategies. The specific effects of these control strategies on free-base nicotine and their applicable conditions are summarized in Table 1. Owing to substantial differences in product type, experimental conditions, puffing regime, and outcome indicators across studies, the available quantitative results were tabulated for structured comparison rather than pooled for meta-analysis.

From the perspective of trade-off analysis, each individual approach entails side effects that are difficult to reconcile. Lowering free-base nicotine through acid addition may simultaneously impair combustibility and increase CO yield [40,60,61]. Promoting nicotine release via glycerol addition may be accompanied by the formation of carbonyl compounds [55]. Increasing moisture content reduces nicotine release, yet also leads to elevated CO yield and incomplete combustion [60,61,62]. These trade-offs indicate that independent regulation of a single parameter is unlikely to simultaneously meet the multiple objectives of reducing irritation, maintaining impact, and controlling harmful constituents; multi-factor synergistic control is therefore an inevitable technical requirement. Based on the above analysis, the selection and improvement directions of internal control strategies for different products under different control objectives are summarized in Table 2.

## 4. Control of Nicotine Release by External Functional Sustained-Release Materials

Internal control strategies can modulate nicotine release by altering nicotine speciation and aerosol carrier properties. However, their ability to precisely control the release rate and release amount remains limited. Moreover, their efficacy can be affected by variations in tobacco composition and puffing conditions. By contrast, external functional sustained-release materials intervene in the nicotine mass-transfer process at the molecular-interaction level, enabling more precise nicotine control through targeted design. These technical pathways can be divided into two main categories. The first is source-loaded controlled release, in which nicotine is pre-loaded onto functional materials through adsorption or inclusion complexation. The nicotine-loaded materials are then incorporated into tobacco feedstocks for reconstituted tobacco sheet preparation, thereby achieving gradient sustained release from the source of nicotine. The second is process-optimized controlled release, in which functional materials are placed in the smoke flow path, such as cooling sections or filter cavities, to selectively adsorb and desorb nicotine from the smoke stream, thereby improving puff-by-puff nicotine delivery stability. These two pathways correspond to “source-end control” and “downstream control” along the full nicotine release chain and constitute distinct technical strategies for controlled release. Current research has focused predominantly on heated cigarette systems, with the core objective of improving puff-by-puff nicotine release stability. The functional materials investigated are mainly concentrated in three categories: bacterial cellulose (BC)-based materials, metal–organic framework (MOF) composites, and porous mineral materials. Mechanistically, the interactions between these material structures and nicotine molecules primarily involve hydrogen bonding and π–π stacking.

### 4.1. Bacterial Cellulose-Based Sustained-Release and Porous Materials

Bacterial cellulose (BC), a high-purity nanocellulose material produced by microbial fermentation, possesses a three-dimensional network structure and a high specific surface area [64,65]. Similar cellulose-based porous materials have also been explored for nicotine control. Zeng et al. [66] developed a cellulose nanofiber–SiO_2_ hybrid aerogel filter, which exhibited strong adsorption capability toward nicotine and other smoke components due to its hierarchical porous structure. Its hydroxyl groups can form hydrogen bonds with nicotine molecules, while its complex network structure impedes nicotine diffusion, making it a suitable carrier for constructing nicotine sustained-release systems. Zhou et al. [67] prepared BC-loaded tobacco leaves using two methods: in situ BC growth and backfilling with concentrated tobacco waste liquor. These methods yielded in situ-grown BC tobacco leaves and backfilled BC tobacco leaves, respectively. The results showed that the backfilled BC tobacco leaves exhibited better thermal stability, lower puff-by-puff nicotine release variability, and a slower dissolution release rate. Moreover, this backfilling approach reduces production costs and enables the high-value utilization of tobacco waste resources. To further stabilize nicotine release, the researchers prepared citric acid–bacterial cellulose (CA-BC) aerogels through chemical crosslinking. This material forms a uniform, clustered three-dimensional network that provides stable binding sites for nicotine and retards the release process through a diffusion barrier effect, thereby exhibiting stronger sustained-release properties than backfilled BC tobacco leaves. Puff-by-puff heating tests at 250 °C demonstrated that the CA-BC aerogel with a 10% crosslinking ratio achieved a puff-by-puff nicotine release variability of 8.55%, substantially lower than the 26.18% observed for backfilled BC tobacco leaves. For nicotine dissolution release, the sustained-release performance of the CA-BC aerogel improved by 35.6–41.2% relative to backfilled BC tobacco. Furthermore, when nicotine loading increased from 0.2% to 2.0%, the thermal release variability of the CA-BC aerogel decreased by 37.2%, and the dissolution rate coefficient decreased by 23.8%, indicating that higher nicotine loading favors a more stable release system.

### 4.2. MOF-Based Composite Controlled-Release Materials

Building on BC-based studies, researchers further combined metal–organic frameworks (MOFs) with BC to obtain materials with enhanced interactions with nicotine. MOFs possess precisely tunable pore structures. The pore sizes of certain MOFs are close to the molecular dimensions of nicotine, enabling nicotine inclusion complexation and sustained release through a spatial confinement effect. β-Cyclodextrin (β-CD) is a cyclic oligosaccharide composed of seven glucose units. It has a hydrophobic inner cavity and a hydrophilic outer surface, enabling it to form inclusion complexes with various guest molecules.

Su et al. [68,69] prepared a β-CD-based metal–organic framework (β-CD-MOF) and introduced it into a BC culture medium together with a concentrated tobacco extract, yielding a tobacco BC@β-CD-MOF composite. Structural characterization demonstrated that the composite retained the fibrous network structure of BC, with β-CD-MOF particles uniformly embedded within it. This structure markedly increased the specific surface area and porosity of the material and provided additional binding sites for nicotine loading and sustained release. In terms of thermal release performance, the β-CD-MOF@tobacco BC composite exhibited favorable sustained-release properties. Compared with the control material without MOF, the composite delayed nicotine release by 1.055 min and increased the proportion of nicotine in the total release by 5.8%. In pyrolysis experiments, the proportion of nicotine released from the β-CD-MOF@tobacco BC composite reached 39.6%, compared with 33.4% for the control material, indicating stronger nicotine loading and controlled-release capability. Furthermore, when the β-CD-MOF@BC composite was applied in the preparation of novel reconstituted tobacco sheets, its sustained-release performance remained evident. Moreover, the novel reconstituted tobacco sheets released a greater variety of aroma components after pyrolysis, which may contribute to improved sensory quality during consumption.

### 4.3. Porous Mineral-Based Functional Materials

Unlike BC-based and MOF-based composite materials, which primarily achieve source-end sustained release of nicotine through adsorption, inclusion complexation, and diffusion retardation, porous mineral-based functional materials mainly function through cooling and selective adsorption. They indirectly control nicotine release by modulating the thermal environment and component distribution of the smoke. Li et al. [70] prepared a porous mineral material with cooling and selective adsorption functions using palygorskite powder as the matrix, together with glass powder, alumina fiber, and polyvinyl alcohol as auxiliary materials, through a mixed calcination method. Under the optimized formulation (palygorskite powder, 3 g; glass powder, 1.5 g; alumina fiber, 1.5 g; polyvinyl alcohol, 1.20 g; calcination at 1200 °C for 2 h), the material achieved a specific surface area of 10.51 m^2^/g and developed abundant mesopores centered at 3.83 nm. It reduced the maximum smoke temperature of heated cigarettes to 45.16 °C, which was 5.79 °C lower than that of a commercial cooling reference material. The control effect of this material on nicotine release is manifested primarily through two synergistic mechanisms: cooling and selective adsorption. In terms of cooling, molecular simulation confirmed that the high specific surface area and abundant micropores of the material increase the thermal contact area between the hot smoke and the material matrix. These structural features also enhance convective heat transfer between the fluid and particles. Together, these effects enable efficient and stable cooling. In terms of selective adsorption, the material reduced nicotine adsorption by 55–73% compared with the control material, thereby substantially minimizing unproductive nicotine retention in the cooling section and allowing more nicotine to remain in the smoke for effective delivery. Meanwhile, the glycerol carrier was retained to a greater extent in the smoke, which may help improve mouthfeel during puffing. This selective control characteristic of “low nicotine adsorption and high glycerol retention” distinguishes the material from conventional adsorbents that simply trap smoke components, making it better suited to the core requirement of stable nicotine delivery in heated cigarettes.

### 4.4. Design and Selection Strategies for External Functional Sustained-Release Materials

Current research on external functional sustained-release materials is limited in scope and remains insufficiently developed (Table 3). Moreover, investigations into the safety and human health impacts of these materials under heating conditions are notably lacking. These areas represent important directions for further advancement in this field.

- Expanding the research system of novel nicotine sustained-release materials. Materials science is advancing rapidly, with a wide range of new functional materials being developed. Covalent organic frameworks (COFs), for instance, offer structural stability, tunable porosity, and excellent adsorption performance, making them highly compatible with nicotine controlled-release scenarios; such novel materials provide considerable scope for further investigation. Meanwhile, emphasis should be placed on elucidating the interaction mechanisms between these materials and nicotine molecules, as well as other components in the smoke environment, to substantially enhance the efficiency of targeted material design. Furthermore, the compatibility of these materials in industrial cigarette production should be validated, thereby addressing the challenges of bridging laboratory-scale technologies to large-scale manufacturing;
- Strengthening human safety research on these materials. Compared with ordinary industrial materials, materials used for nicotine controlled release are subject to more stringent safety requirements, the core of which is ensuring human health safety, particularly with regard to inhalation exposure. Therefore, during material development, relevant safety testing must be conducted in parallel. The focus should be on evaluating whether the materials release harmful substances under the heating conditions of cigarette operation, and whether material particles can migrate with the smoke aerosol into the human body and pose health risks. A systematic toxicological safety evaluation of the materials should be completed.

## 5. Comparative and Synergistic Analysis of Internal and External Controlled-Release Strategies

The preceding sections have elaborated on the mechanisms and research progress of two distinct systems: internal control within tobacco systems and external controlled release via functional materials. The former modulates nicotine release in situ by altering nicotine speciation and aerosol carrier properties, whereas the latter intervenes in the mass transfer process—through adsorption, diffusion retardation, and related mechanisms—to regulate the release rate and stability of nicotine. These two systems are complementary in their control rationale, yet they differ in their applicable scope, technological maturity, and the challenges they face. This section provides a horizontal comparison of the two systems, analyzes their synergistic mechanisms, and discusses adaptive application strategies tailored to the controlled-release requirements of different tobacco products.

### 5.1. Core Characteristics of Internal and External Controlled-Release Systems

Table 4 presents a systematic comparison of the two systems across five dimensions: control site, mechanism of action, main advantages, current limitations, and applicable product types.

The comparison indicates that internal control acts at the source of nicotine release, determining the baseline level and overall trend of release, and thus occupies a dominant position in industrial applications. External material-based controlled release, by contrast, directly intervenes in the nicotine mass transfer process, retarding release through physical interception or diffusion retardation, and outperforms internal control in the precision of puff-by-puff release stability. These two systems are not mutually exclusive but rather stand in a foundation–refinement relationship: internal control establishes the fundamental release framework, within which external materials perform fine-tuning.

### 5.2. Synergistic Strategies of Internal and External Controlled-Release Systems

A single control system alone is unlikely to simultaneously meet the multiple objectives of release precision, sensory quality, production compatibility, and safety; their synergistic combination represents a noteworthy direction for further development in this field. Based on existing research, the synergistic effects are manifested primarily at two levels.

- Synergy between the basal environment and control precision. Internal control can pre-adjust nicotine speciation and the matrix environment—for instance, by modulating the free-base nicotine fraction through organic acid addition—thereby providing a relatively stable starting point for external materials. When the nicotine speciation and carrier state of the tobacco matrix are already within a favorable range, the subsequent fine-tuning of diffusion rate and puff-by-puff stability by external materials is likely to be more effective than when applied to an unoptimized matrix. This “internal control as foundation, external control for precision refinement” approach holds promise for improving the overall consistency and predictability of controlled release;
- Synergy between quality optimization and risk control. Internal control measures such as increasing moisture content or adding acids, while reducing nicotine release or irritation, may concurrently elevate CO release or impair combustibility [60,61]. External porous mineral materials, with their ability to adsorb certain low-molecular-weight harmful constituents from smoke while retaining nicotine and glycerol to a greater extent [70], offer the potential to mitigate these side effects. Furthermore, external sustained-release materials can enhance the effective delivery efficiency of nicotine; when these materials can achieve relatively stable release at lower nicotine loadings, the internal nicotine content of the tobacco matrix can be appropriately reduced, thereby indirectly lowering the potential for harmful constituent release in the smoke.

### 5.3. Synergistic Controlled-Release Strategies for Different Tobacco Products

Different types of tobacco products differ in their puffing regimes, product structures, and core controlled-release requirements, and the appropriate synergistic strategies vary accordingly.

- For conventional combustible cigarettes, the core requirements lie in reducing smoke irritation and lowering harmful constituent release. Internal strategies may include the addition of volatile organic acids or moderate pH lowering to reduce the free-base nicotine fraction, while controlling cut tobacco moisture content within an appropriate range to balance combustion efficiency and nicotine release. On this basis, functional materials can be introduced into the filter section as an external auxiliary approach, utilizing their adsorption and diffusion retardation functions to further smooth puff-by-puff release and reduce certain harmful constituents;
- For heated cigarettes, the core challenges lie in relatively low nicotine delivery efficiency and substantial puff-by-puff release fluctuation. At the internal level, lactic acid can be used to modulate free-base nicotine speciation, and the glycerol addition ratio can be optimized to improve aerosol carrier efficiency. At the external level, functional materials can be embedded into the reconstituted tobacco matrix, utilizing chemical crosslinking networks or micro/nano-pores to retard nicotine release; additionally, porous mineral materials can be incorporated in the cooling section to lower smoke temperature or achieve other functions;
- For e-cigarettes, the core requirement is to balance a high nicotine concentration with low inhalation irritation. Existing studies have demonstrated that adding organic acids to form nicotine salt systems can reduce the free-base nicotine fraction to approximately 0.1, thereby lowering irritation while maintaining a high total nicotine concentration. Research on external materials in this area remains limited; potential directions for exploration include the development of temperature-sensitive or pH-responsive controlled-release materials to improve the stability of nicotine release under varying power conditions.

**Figure 2:**
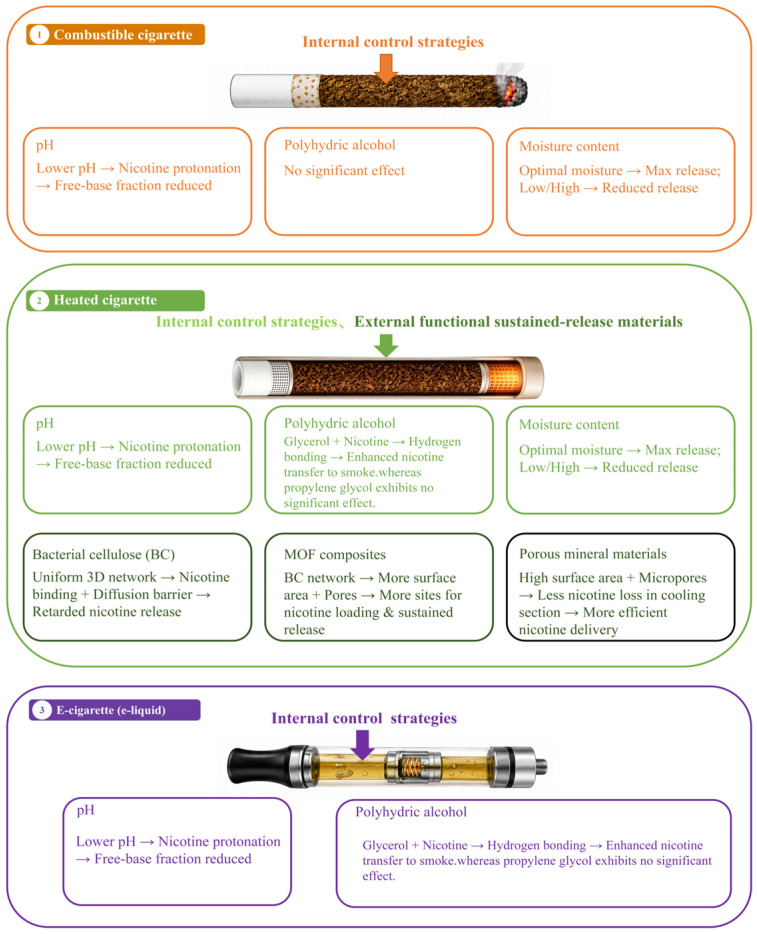
Product-specific integration of internal control strategies and external functional sustained-release materials for nicotine release control in tobacco products.

The product-specific integration of internal control strategies and external functional sustained-release materials is summarized in Figure 2.

## 6. Conclusions

This review has systematically elucidated three core mechanisms governing nicotine controlled release—nicotine speciation conversion, heat and mass transfer, and mass transfer retardation—clarified the complementary relationship between internal control and external materials, and established a full-chain technological framework for nicotine controlled release.

Internal control, primarily employing organic acids and pH, polyols, and moisture content, modulates nicotine release by altering nicotine speciation, aerosol carrier properties, and heat and mass transfer conditions, representing the most technologically mature and widely applied approach in current practice. External functional materials, represented by bacterial cellulose-based materials, MOF composites, and mineral-based materials, intervene in the nicotine mass transfer process through mechanisms including physical retardation, chemical adsorption, molecular inclusion complexation, and environmental synergy, offering an important precision advantage in improving puff-by-puff release stability. These two systems are not mutually exclusive but rather stand in a foundation–refinement relationship.

Based on the existing research progress and current limitations, future research may focus on the following directions.

- Moving from single-factor control to multi-factor synergy. Current research has predominantly focused on the effects of individual factors; the interactive effects among organic acids, glycerol, moisture content, and other parameters remain insufficiently understood, and systematic exploration of the matching principles between internal parameters and external materials is also lacking. Establishing quantitative multi-factor coupled control models represents a fundamental issue that must be addressed for this field to progress from empirical formulation toward rational design;
- Integrating molecular simulation and machine learning into the material development workflow. The screening of external sustained-release materials currently relies predominantly on experimental trial and error. Molecular simulation can reveal, at the atomic scale, the interaction modes between nicotine molecules and surface functional groups of materials, as well as their diffusion behavior within pores, thereby providing a microscopic basis for material structural design. Machine learning can establish predictive models linking material structural features with controlled-release performance, reducing reliance on empirical judgment and accelerating the discovery of high-performance sustained-release materials;
- Establishing a comprehensive effect evaluation system for controlled-release technologies. While modulating nicotine release behavior, control measures may exert concomitant effects on other smoke constituents. A crucial step in moving these technologies from functional validation to product application is to incorporate harmful constituent analysis and toxicological evaluation throughout the entire development process. This will provide a health-risk-based scientific perspective for selecting control strategies.

## Figures and Tables

**Table 1:** Effects of internal control strategies on free-base nicotine release.

Control Strategies	Effect on Free-Base Nicotine	Applicable Product Type	Mechanism and Key Points	References
Organic acid/pH control				
Addition of volatile organic acids (acetic acid, levulinic acid) and semi-volatile lactic acid	Markedly decreased (68.7–75%)	Combustible cigarettes, heated cigarettes	Entry of organic acids into aerosol during combustion/heating and in situ protonation	[10,51]
Addition of non-volatile organic acids (citric acid, tartaric acid, malic acid)	No appreciable effect	Heated cigarettes	Insufficient volatility of non-volatile acids under heating conditions, preventing interaction	[51]
Citric acid (freeze-drying, filter addition)	Decreased (36.8%)	Combustible cigarettes	Non-volatile acid with increased specific surface area via specialized addition method enables direct contact with and protonation of nicotine along the smoke path	[50]
Lowering system pH (acid addition)	Markedly decreased (50.5%)	All tobacco product types	pH directly governs the free-base/protonated equilibrium; acid addition protonates nicotine, reducing the free-base fraction	[14,40]
Increasing system pH (base addition)	Markedly increased (164.8%)	Oral tobacco products	Elevated pH promotes nicotine deprotonation, enhancing oral absorption efficiency	[40]
Polyol control				
Glycerol addition (heated cigarettes, e-cigarettes)	Markedly increased (10–46%)	Heated cigarettes, e-cigarettes	Glycerol, with three hydroxyl groups, forms hydrogen bonds with nicotine and functions as an aerosol carrier, facilitating nicotine transfer from the matrix to the smoke	[55,57]
Glycerol addition (combustible cigarettes)	No appreciable enhancement	Combustible cigarettes	Glycerol is largely decomposed at high combustion temperatures, substantially diminishing its promoting effect on nicotine release	[54]
Propylene glycol addition	No appreciable effect	E-cigarettes, heated cigarettes	Propylene glycol, with only two hydroxyl groups, interacts more weakly with nicotine than glycerol, serving primarily as a humectant and atomization aid	[56]
Moisture content control				
Increasing moisture content (combustible cigarettes)	Decreased (12.6–30.4%)	Combustible cigarettes	Water vaporization absorbs heat, reducing combustion temperature and retarding nicotine thermal desorption, while concurrently increasing CO and tar yields	[60,61,62]
Moisture content equilibration (heated cigarettes)	Optimal range observed (34.2–39.9%)	Heated cigarettes	Too low moisture content leads to uneven heat transfer, whereas too high moisture content inhibits aerosol entrainment; under 50% relative humidity equilibration, nicotine transfer efficiency is highest	[63]

**Table 2:** Selection and improvement directions of internal control strategies for different tobacco products.

Product Type	Control Objective	Recommended Control Strategy	Advantages and Limitations	Future Development Directions
Combustible cigarette	Reducing smoke irritation	Addition of volatile organic acids or moderate pH lowering	Effectively reduces free-base nicotine by directly modulating nicotine speciation; however, excessive acid addition may impair combustibility and elevate CO, and non-volatile acids show limited efficacy under conventional addition methods	Exploring organic acid modulators with moderate volatility and good thermal stability; investigating the trade-off boundaries between acid dosage, combustibility, and CO release
	Delaying nicotine release rate	Moderately increasing cut tobacco moisture content	Offers the advantages of in-process implementation at low cost; yet excessively high moisture content markedly elevates CO and causes incomplete combustion, and the regulatory range is limited	Combined use of moisture content and pH control (moderate acid addition under high moisture content to compensate for combustibility issues); synergistic application with filter materials, superimposing physical adsorption on moisture content regulation
Heated cigarettes	Reducing irritation	Addition of organic acids with moderate boiling points, such as lactic acid	Lactic acid contains both carboxyl and hydroxyl groups, enabling effective protonation of nicotine even under heating conditions; however, high-boiling-point non-volatile acids such as citric acid exert only minimal control in this system, and the choice of organic acids is constrained by the heating temperature	Screening or designing organic acid modulators with boiling points matched to the operating temperature of heated cigarettes; utilizing organic acid–glycerol combinations to achieve synergistic optimization of irritation and nicotine release
	Increasing nicotine release	Increasing the glycerol addition ratio	Glycerol forms hydrogen bonds with nicotine and acts as a carrier, producing a marked release-promoting effect; however, the effect saturates beyond 10% addition, excessive glycerol may generate carbonyl compounds, and propylene glycol does not share this function	Developing glycerol–polyol blended systems to balance release promotion with reduced harmful compound formation; investigating the quantitative relationship of synergistic glycerol–nicotine release to guide addition ratio optimization
E-cigarettes	Balancing high nicotine concentration with low irritation	Addition of organic acids to form nicotine salts	Maintains high total nicotine concentration while reducing the free-base nicotine fraction to approximately 0.1, thereby lowering inhalation irritation; however, the organic acid-to-nicotine molar ratio is the critical parameter, with a narrow control window.	Developing rapid optimization methods for acid-to-nicotine ratios; evaluating safety differences among nicotine salts formed with various organic acids; exploring the potential of multi-acid blended systems.
	Enhancing nicotine delivery efficiency	Optimizing the glycerol ratio and puffing regime	Glycerol serves as the primary aerosol matrix; increasing its ratio enhances nicotine delivery, and square-wave puffing increases total release. However, excessive glycerol impairs atomizer performance, and puffing parameters are tightly coupled with product design.	Establishing a ternary relationship model linking glycerol ratio, power, and nicotine release; developing standardized parameter systems that balance delivery efficiency with device compatibility.

**Table 3:** Effects of external functional sustained-release materials on nicotine release control.

Control Strategy	Effect on Nicotine Sustained-Release Performance	Applicable Product Type	Mechanism and Key Points	References
Bacterial cellulose (BC)	Improved by 35.6–41.2%	Heated cigarettes	Forms a uniform clustered three-dimensional network structure, providing stable binding sites for nicotine and retarding the release process through a diffusion barrier effect	[67]
Metal–organic framework (MOF) composites	The proportion of nicotine in the total release increased by 5.8%.	Heated cigarettes	The fibrous network structure of BC increases the specific surface area and porosity of the material, providing more active sites for nicotine loading and sustained release	[68,69]
Porous mineral materials	Nicotine adsorption decreased by 55–73%	Heated cigarettes	The high specific surface area and abundant micropores substantially reduce unproductive nicotine retention in the cooling section, allowing more nicotine to remain in the smoke for effective delivery	[70]

Note: Values indicate percentage decreases or increases compared with the control group.

**Table 4:** Comparison of core characteristics between internal and external controlled-release systems for nicotine release.

Comparison Dimension	Internal Control System	External Functional Material Controlled-Release System
Control site	(1) Acts within the tobacco raw material, i.e., the cut tobacco or reconstituted tobacco matrix; (2) functions as front-end control, influencing the initial state of nicotine release	(1) Can cover the entire release chain; (2) categorized into core-loaded type (front-end) and filter/cooling section control type (back end)
Mechanism of action	(1) Alters the free-base nicotine fraction through protonation–deprotonation equilibrium; (2) influences the transfer efficiency of nicotine into the aerosol via carrier effects; (3) modifies the thermal behavior and mass transfer conditions of the system through moisture content	(1) Retards nicotine diffusion through physical blockage by porous networks; (2) enhances adsorption via hydrogen bonding between functional groups and nicotine molecules; (3) indirectly regulates nicotine volatilization through the cooling function of mineral materials
Main advantages	(1) Relatively high compatibility with existing production processes; (2) does not alter the original product structure, with a relatively manageable impact on sensory quality; (3) low raw material costs and a foundation for large-scale implementation	(1) More targeted regulation of nicotine release rate; (2) surpasses internal control in the precision of improving puff-by-puff release stability; (3) can impart additional functions such as aroma enhancement and cooling
Current limitations	(1) Limited capacity for precise modulation of the release rate; (2) some control approaches may be accompanied by increased tar or CO release; (3) control efficacy is constrained by product type and puffing regime	(1) Research on compatibility with production processes remains insufficient; (2) the preparation cost of some materials is relatively high; (3) scale-up application pathways and safety assessment systems require further development
Applicable products	Suitable for all categories of tobacco products	(1) The core-loaded type is better suited to the reconstituted tobacco system of heated cigarettes; (2) the process control type can be adapted to the filter or cooling section of various cigarette types

## Data Availability

No new datasets were generated for this review. The search strategy, screening framework, and extracted summary information supporting the conclusions of this manuscript are available from the corresponding author upon reasonable request.

## Abbreviations

The following abbreviations are used in this manuscript:

BC	bacterial cellulose
CA-BC	citric acid–bacterial cellulose
MOF	metal–organic framework
β-CD	β-Cyclodextrin
β-CD-MOF	β-cyclodextrin-based metal–organic framework
COF	covalent organic framework
ISO	International Organization for Standardization
HCI	Health Canada Intense (puffing regime)
CO	carbon monoxide
